# EEG-Based Emotion Recognition by Exploiting Fused Network Entropy Measures of Complex Networks across Subjects

**DOI:** 10.3390/e23080984

**Published:** 2021-07-30

**Authors:** Longxin Yao, Mingjiang Wang, Yun Lu, Heng Li, Xue Zhang

**Affiliations:** 1 School of Electronic and Information Engineering, Harbin Institute of Technology at Shenzhen, Shenzhen 518055, China; 18631149461@163.com (L.Y.); 19s152117@stu.hit.edu.cn (H.L.); 13115501669@163.com (X.Z.); 2 School of Computer Science and Engineering, Huizhou University, Huizhou 516007, China

**Keywords:** emotion recognition, complex network, network entropy measure, machine learning

## Abstract

It is well known that there may be significant individual differences in physiological signal patterns for emotional responses. Emotion recognition based on electroencephalogram (EEG) signals is still a challenging task in the context of developing an individual-independent recognition method. In our paper, from the perspective of spatial topology and temporal information of brain emotional patterns in an EEG, we exploit complex networks to characterize EEG signals to effectively extract EEG information for emotion recognition. First, we exploit visibility graphs to construct complex networks from EEG signals. Then, two kinds of network entropy measures (nodal degree entropy and clustering coefficient entropy) are calculated. By applying the AUC method, the effective features are input into the SVM classifier to perform emotion recognition across subjects. The experiment results showed that, for the EEG signals of 62 channels, the features of 18 channels selected by AUC were significant (*p* < 0.005). For the classification of positive and negative emotions, the average recognition rate was 87.26%; for the classification of positive, negative, and neutral emotions, the average recognition rate was 68.44%. Our method improves mean accuracy by an average of 2.28% compared with other existing methods. Our results fully demonstrate that a more accurate recognition of emotional EEG signals can be achieved relative to the available relevant studies, indicating that our method can provide more generalizability in practical use.

## 1. Introduction

The basic abilities of people in social communication include accurately distinguishing emotions and making reasonable responses [1]. With the ability of recognizing emotions, machines could think like humans, perceive human emotional states, and make rational responses [2]. However, the relevant research focuses more on logic and ignores the importance of emotions related to human–computer interfaces (BCI) [3,4]. Current emotion recognition methods are mainly based on non-physiological signals and physiological signals. Non-physiological studies mainly contain speech signals [5], facial expressions [6], body postures [7], and gestures [8]. On the other hand, physiological signals excellently reflect the functions of humans, with the advantages of objectivity and accuracy. Common physiological signals are also diverse, and include electroencephalogram (EEG) [9], electromyogram (EMG) [10], and electrocardiogram (ECG) [11], etc. Among the above physiological signals, EEG signals can be obtained from the cerebral cortex using noninvasive devices, with the advantages of being direct, noninvasive and safe. Emotion states can be directly reflected by EEG signals related to corresponding brain regions. Based on the above case, emotion recognition based on EEG signals has attracted more and more attention due to its characteristics of being safe, noninvasive and intuitive [12,13,14,15].

Relevant studies on affective classification based on EEG have faced many scientific challenges in the past decade. First, in the same context, when the emotional incentives are different, due to differences in gender, age, education, cultural background, etc., people differ in their emotional expression, and each person’s emotional experience is also different. Individual differences directly affect the processing results of emotion recognition in the same environment, which leads to a lack of generalizability in the existing emotion recognition models [16,17]. Furthermore, relevant research mostly suffers from large amounts of calculation and being time-consuming, using deep learning pattern classification. In addition, the EEG signals corresponding to the emotion-related sections of the brain offer rich spatial information, because of different areas of the cerebral cortex being associated with complex emotions [18]. However, traditional features in the existing studies are limited by time, and lack the spatial characteristics of EEG signals. Therefore, in order to address these challenges, it is of great research value to develop an EEG emotion recognition technique across subjects, that includes time and spatial domain information.

In the past decade, several methods based on complex network theory for analyzing EEG signals, in the form of time series with characteristics of nonlinearity and nonstationary, were proposed [19,20]. A complex network is a reasonable and effective method for studying nonlinear time series and nonlinear dynamic systems [21]. A nonlinear time series can be reasonably transformed into a complex network [22], whereby the statistical indices of the network topology structure can be analyzed to determine the properties of the nonlinear time series. As a classical method to construct complex networks, the visibility graph method is based on visual conditions, thus extending the research platform of nonlinear time series to the level of a complex network. By directly connecting the amplitude of the time series, the line meeting the visual condition is retained as the connecting edge of the complex network, such that the time series is transformed into a corresponding complex network composed of nodes and connecting edges [23]. Since the visibility graph method of constructing complex networks has the property of remaining unchanged under affine changes, its topological properties can effectively describe the characteristics of EEG signals. The visibility graph method is used to present the constructed network in the domains of time and space because it contains multiple network entropy measures [24,25].

In this paper, we developed a cross-subject emotion recognition method that contains rich EEG time and spatial domain information to identify positive and negative emotions from EEG signals in a cross-subject situation. The purpose of our work is to enhance the robustness of the cross-individual emotion recognition method, exploiting the fused network entropy measures of complex networks. The main innovations of our emotion recognition method are as follows: (a) The complex network related to brain emotion was constructed using the visibility graph method from EEG signals, and rich spatial information of brain signals was retained. (b) Network entropy measures were derived from the complex networks. We applied NDE and CCE, which allow for characterizing complex networks globally and locally as a performance index of brain network analysis, to reveal the complexity and dynamic behavior of the brain experiencing different emotions. (c) The cross-subject emotion training method based on the SEED dataset was used to overcome individual differences, which thus makes emotion recognition more universal and generalized. The main contributions of this paper are outlined below.

Firstly, we exploit the EEG features of network entropy measures based on time and spatial domains as effective emotion recognition patterns. In contrast to the traditional EEG emotional patterns, the adopted EEG emotional features can extract the local and global features from complex networks associated with brain networks. Secondly, we develop an excellent emotion recognition method by taking advantage of a machine learning model across individuals, which effectively enhances robustness. Finally, our experimental results fully demonstrate that the proposed method is able to achieve a better performance in cross-subject emotion recognition, with a higher accuracy than other existing methods. 

The organization of this paper is as follows: Section 2 describes the visibility graph method and the extraction of network entropy measures. Section 3 presents the data analysis and experimental results. In Section 4, we discuss the experimental results in comparison with recently reported studies. Lastly, the article is concluded in Section 5.

## 2. Materials and Methods

**Figure 1:**
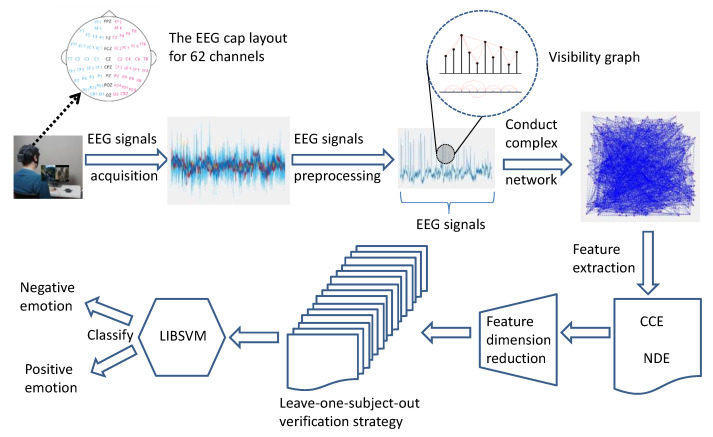
Framework of our proposed emotion recognition model.

The framework of our proposed emotion recognition model is shown in Figure 1, comprising several processes. First, for the subjects, video stimuli were used to elicit emotional reactions and EEG data were recorded. When the preprocessing of EEG signals was completed, the EEG signals were mapped as a complex network using the visibility graph method. Next, two features (NDE and CCE) were calculated from the complex network. In addition, the AUC values of each channel were calculated, and the effective channels were selected according to the defined criterion (AUC = 0.8) to classify emotions. The features of the selected channels were fused to a feature vector sent to a machine learning model. Lastly, the support vector machine (SVM) was used as a pattern classifier to recognize different emotions.

### 2.1. Experimental Dataset

Our study used an open dataset for analysis, namely, SEED (SJTU Emotion EEG Dataset) [15]. This dataset includes 15 Chinese subjects (7 males and 8 females; mean value: 23.27; STD: 2.37). A total of 15 Chinese film clips (positive, neutral, and negative emotions) were selected as the subjects’ emotional stimuli, with each film lasting about 4 min. A total of three groups of experiments were conducted, with each subject participating in 15 trials in each case. Three kinds of emotions were induced in each subject, i.e., positive, neutral, and negative emotions, through an emotional film clip. Therefore, each subject participated in three experiments of 15 trials each, i.e., the subjects conducted 45 (15 × 3) trials using the SEED dataset. For more information about the SEED database, please refer to http://bcmi.sjtu.edu.cn/seed/ (accessed on 25 July 2021). In this study, we used EEG signals corresponding to trials of positive, neutral, and negative emotions to distinguish among the three types of emotions. We intercepted EEG signals from SEED for 30 s of data, extracted from the middle portion of each trial, i.e., from 60 s to 90 s.

### 2.2. Constructing Complex Networks Using Visibility Graph from EEG Signals

**Figure 2:**
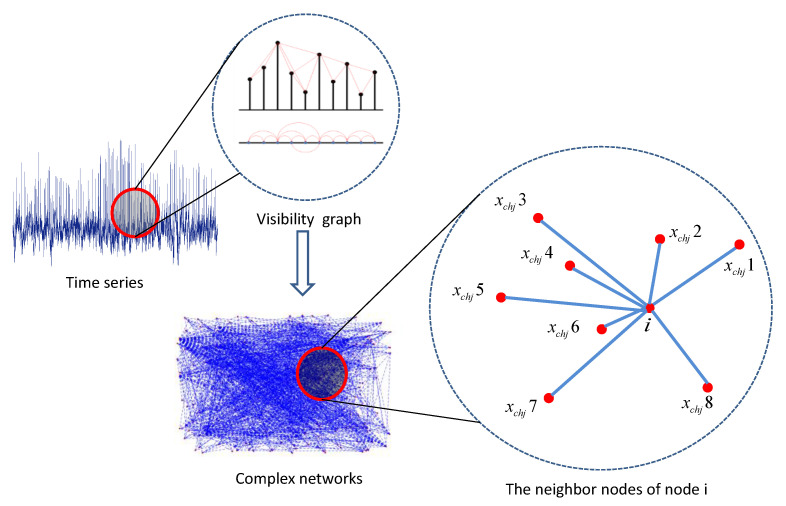
Construction of complex networks using visibility graph derived from EEG signals.

The EEG results are shown in the form of a signal time series, which has nonlinear and nonstationary characteristics. Time series signals can be effectively and quantitatively analyzed using complex network theory, e.g., visibility graphs. EEG signals contain 62 channels, each of which is a time series of *N* points. The main operation method of the visibility graph is to transform the time series into numerical points $\{x_{chj}(i)\},i=1,2,\ldots ,N,j=1,2,\ldots 62$, and each numerical point corresponds to the height of the vertical axis coordinate system, displayed as vertical lines; then, the numerical points are defined as a network, and these network nodes are connected as a function of the numerical points. As shown in Figure 2, node i has neighbor nodes $x_{chj}i=\left(x_{chj}1,x_{chj}2,\ldots ,x_{chj}m-1,x_{chj}m\right)$, 1≤i≤m, where m is the total number of neighbor nodes. The visibility graph method remains unchanged following affine changes; as such, its topological properties can effectively describe the characteristics of a time series.

### 2.3. Fusion of Network Entropy Measures

After constructing the complex network corresponding to the EEG signals, for its comprehensive characterization, we described the spatial characteristics of the brain network as a function of its global and local characteristics using the CCE and NDE of EEG signals for different emotions calculated on each channel. CCE, as a local feature of the network, describes the attribute of local node connectivity in the network, and represents the distribution of the proportion of interconnected neighbor nodes in the whole network [26,27,28]. CCE also represents the clustering state of nodes in the network. In a network with a large number of nodes, the nodes form high-density connections, which lead to local clustering. In the complex network corresponding to the EEG signals, CCE is defined as

(1) $$\mathrm{CCE}=-\sum_{i}ClogC,$$

(2) $$C=\frac{1}{n}\sum_{i\in N}C_{i}=\frac{1}{n}\sum_{i\in N}\frac{2e_{pq}}{m_{i}\left(m_{i}-1\right)},$$

where $e_{pq}$ is the one-hop connection between neighbor node *p* and node *q*, and $m_{i}$ is the total number of neighbor nodes connecting to node i.

As a global characteristic of the network, NDE describes the neighborhood degree of global nodes in the network, which represents an effective measure of network node integration. NDE is an effective method to evaluate node heterogeneity from the perspective of neighborhood degree. In complex networks, NDE is defined as

(3) $$\mathrm{NDE}=-\sum_{i}p_{i}logp_{i},$$

where $p_{i}$ is the probability description of the nodal degree about node i. This is defined as

(4) $$p_{i}=\frac{d_{i}}{\sum_{j\in N}d_{j}},$$

where $d_{i}$ is the neighbor node connected to node i.

The pseudo code for emotion recognition based on complex networks is presented in Algorithm 1. The sampling rate was 200 Hz. Each data point was used as a network node. The time series was constructed into complex networks using the visibility graph method. The CCE and NDE features were extracted from the complex networks, and both features were set as the feature vector.
**Algorithm 1.** Extraction of emotion features based on complex network**Input**: EEG time series from 15 subjects 1: Initialization 2: M ← the channel number of EEG signals 3: Y ← the nodes of complex networks constructed from EEG signals 4: *HC* ← the number of neighbors of each node 5: *KB* ← the number of hops between nodes 6: *L*(*j*,:) ← sum (HC(*j*,:)) 7: *S*(*j*,:) ← sum (KB(*j*,:)) 8: $m_{i}$ ← the total number of neighbor nodes connecting to node i 9: for *j* = 1 → *M* do begin 10:  for *i* = 1 → Y 11:   $x_{chj}i$ ← $x_{chj}1$, $x_{chj}2$, …, $x_{chj}Y$ 12:  end 13:  ∀*t*, 1 ≤ *t* ≤ $m_{i}\left(j\right)$ do begin 14:   CC(*j*,*t*) ← (1/*t*)*(( (2*KB*(j,t)*)/($S_{Y}$(*j*,1)*($S_{Y}$(*j*,1)−1)))) 15:   CCE(*j,t*) ← −(CC(*j,t*).*(log(CC(*j,t*)))) 16:  ∀*t*, 1 ≤ *t* ≤ $m_{i}\left(j\right)$ do begin 17:   ND(*j,t*) ← (HC(*j,t*))./L(*j*,1) 18:   NDE(*j,t*) ← −(ND(*j,t*).*(log(ND(*h,t*)))) 19: end Output: vector <CCE, NDE>

### 2.4. Feature Dimension Reduction

The redundant information in the feature dimension should be reduced to optimize the feature vector for machine learning. The accuracy of the classification result and the machine learning efficiency can be improved by eliminating invalid feature dimensions and retaining valid feature dimensions. In our work, feature dimensionality reduction was carried out using the AUC method. Aiming at reducing feature dimensionality, this paper first screened 62 channels in the SEED dataset. Then, we selected the most effective channels among the 62 channels. Several feature dimension reduction methods have been introduced, such as paired *t*-test, mutual information, PCA, and AUC. The performance of these feature dimension reduction methods varied. In our work, we used AUC as the feature dimension reduction method, which is defined as the area bounded by the coordinate axis under the ROC curve. Obviously, the value of this area is less than 1. Since the ROC curve is generally above the line y=x, the value range of AUC is between 0.5 and 1. An AUC value closer to 1 denotes the higher authenticity of the test method and a higher application value. The AUC method judges the difference between samples by calculating the area under the ROC curve of multiple samples. After channel screening using the AUC method, the entropy of the network structure of positive and negative emotions derived from EEG signals was calculated for the selected channel to achieve the effect of dimensionality reduction.

### 2.5. Support Vector Machine Classifier

As a machine learning algorithm, the support vector machine is a classification model whose purpose is to find a hyperplane with which to segment samples. The principle of segmentation is to maximize the interval. Each channel of EEG signals was calculated as a network structural feature. We obtained 128-dimensional features by calculating CCE and NDE for 62 channels. Then, we used the leave-one-subject-out verification strategy to specify the test and training sets. The SEED dataset contained 15 subjects, and the data of one subject were used as the testing set, while the data of the remaining subjects were used as the training set; this was repeated for each subject. SVM was used as a classifier of positive and negative emotions or of positive, negative, and neutral emotions. SVM is a kernel-based classifier that can achieve both linear and nonlinear classification via the use of various kernel functions, which differ in their performance. We compared several of the most commonly used existing kernels of SVM, with the results revealing the radial basis function (RBF) as the best performer.

**Figure 3:**
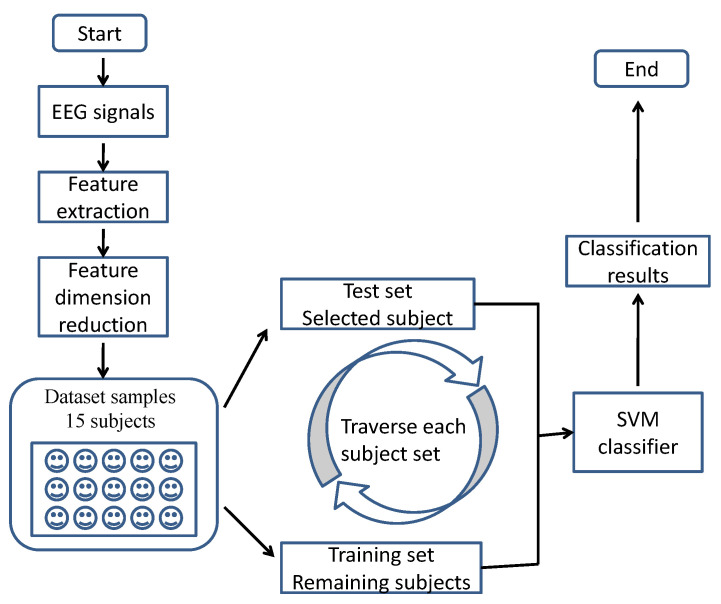
Framework of complex network structure entropy measures for emotion recognition across individuals.

The LIBSVM software package was used for the SVM classifier, along with the RBF kernel. The parameters in the SVM classifier included S, T, C, and other default values. Parameter T was set to 2 and parameter S was set to 0. Parameter C was an optimum value determined through a one-step search of the parameter space ($10^{-3:2}$). The framework of complex network entropy measures for emotion recognition using the SVM classifier is described in Figure 3.

## 3. Results

**Figure 4:**
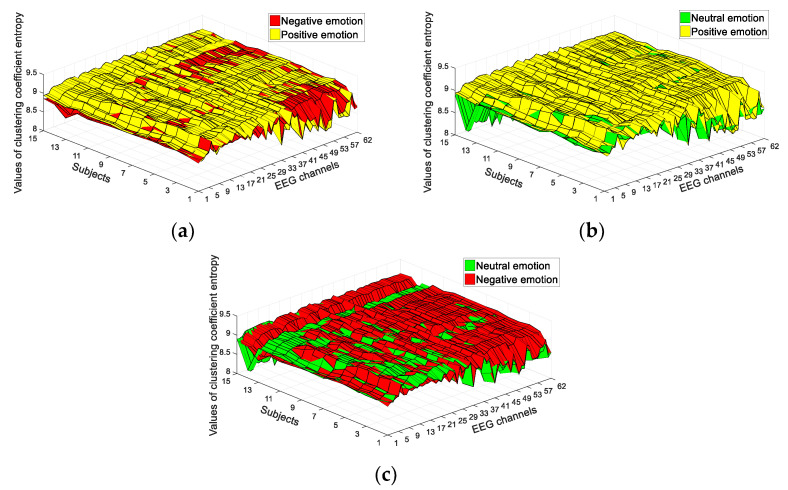
Results of CCE of EEG signals: (**a**) CCE values between positive and negative emotions; (**b**) CCE values between positive and neutral emotions; (**c**) CCE values between negative and neutral emotions.

**Figure 5:**
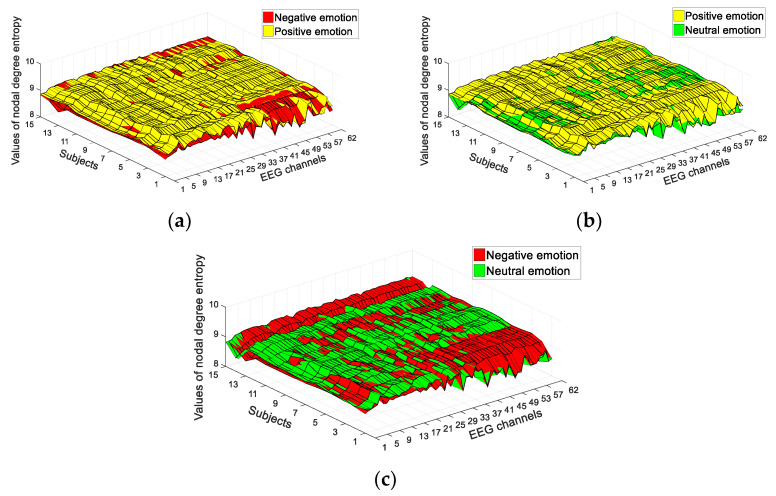
Results of NDE of EEG signals: (**a**) NDE values between positive and negative emotions; (**b**) NDE values between positive and neutral emotions; (**c**) NDE values between negative and neutral emotions.

Firstly, we analyzed the NDE and CCE features of the complex network, which were extracted to classify positive and negative emotions from EEG signals. The time duration of EEG data used in the study was 30 s, selected from the middle of the 62-channel EEG signals (i.e., from 60 s to 90 s). The SEED dataset included 15 subjects and 62-channel EEG signals. We calculated the values of network structural entropy from each channel, thereby obtaining two types of 62-dimensional features, namely, CCE and NDE. Figure 4 shows the results for the CCE of EEG signals in classifying positive and negative emotions, positive and neutral emotions, and negative and neutral emotions. The CCE of most channels presented significant differences. As shown in Figure 4, 68% of the EEG channels exhibited a significant difference between positive and negative emotions (*p* < 0.005), 74% exhibited a significant difference between positive and neutral emotions (*p* < 0.005), and 65% exhibited a significant difference between neutral and negative emotions (*p* < 0.005). The NDE of most channels presented significant differences. As shown in Figure 5, 64% of EEG channels exhibited a significant difference between positive and negative emotions (*p* < 0.005), 69% exhibited a significant difference between positive and neutral emotions (*p* < 0.005), and 58% exhibited a significant difference between neutral and negative emotions (*p* < 0.005). Therefore, according to the results of these two network structural entropies, positive, negative, and neutral emotions could be deduced.

**Figure 6:**
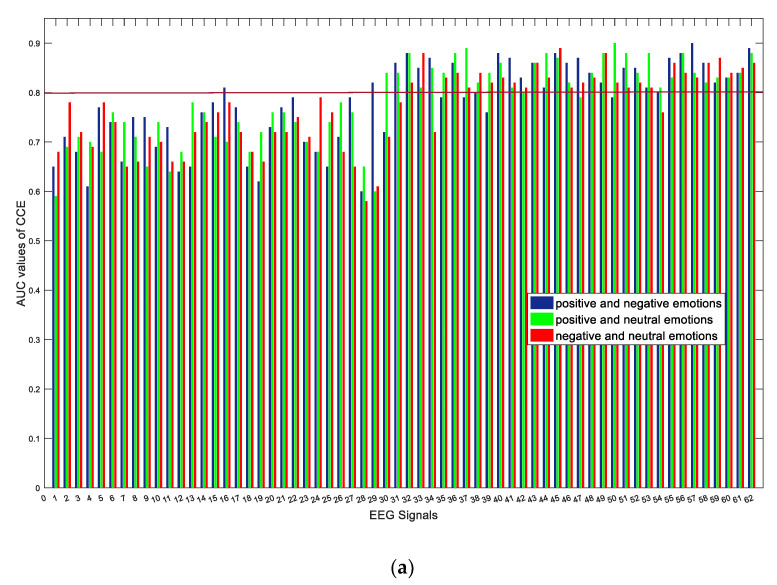

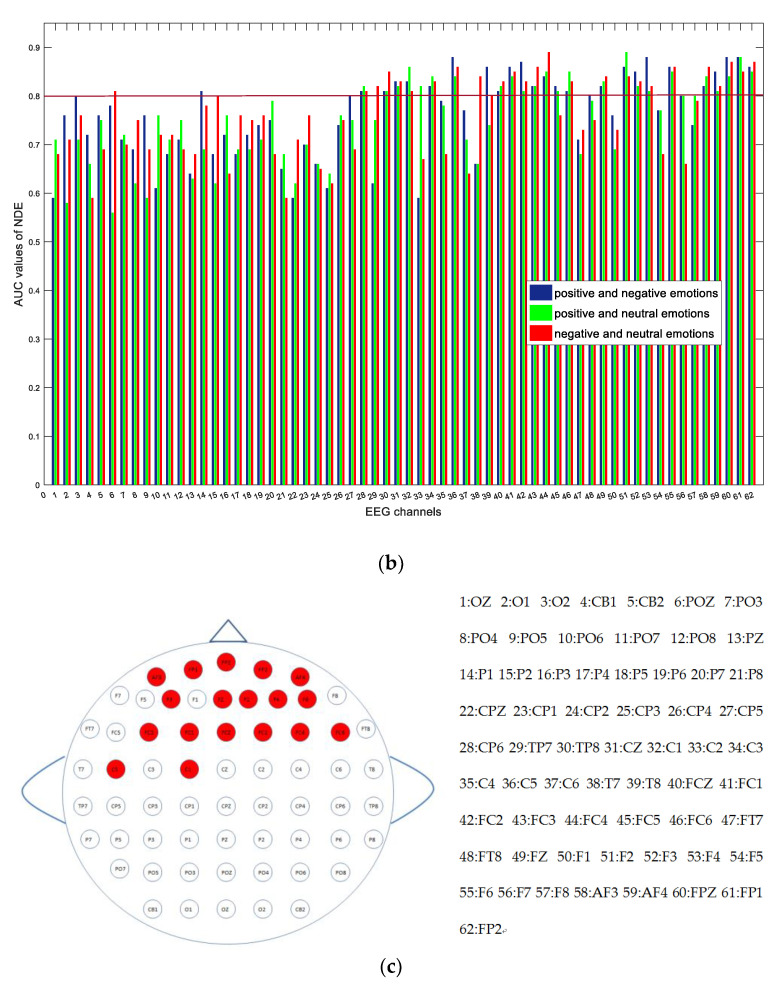
AUC values of each channel: (**a**) AUC values based on CCE; (**b**) AUC values based on NDE; (**c**) selected electrode positions in SEED dataset.

Depending on the emotion, these two network structural entropies presented different discriminative abilities for each EEG channel. We analyzed the contrast in area under the ROC curve for any two kinds of emotions (i.e., various combinations of positive, negative, and neutral emotions). Figure 6 shows the AUC values of structural entropy for the 62-channel EEG network with respect to these combinations. The horizontal axis represents the 62 EEG channels, while the vertical axis represents the AUC values. In our work, we defined the AUC threshold as 0.8, whereby values above this threshold were considered to represent effective channels. Figure 6 also presents the electrode positions for the SEED dataset, wherein the selected channels using network structure entropy and the ROC method are highlighted in red, namely, C1, C5, FCZ, FC1, FC2, FC3, FC4, FC6, FZ, F2, F3, F4, F6, AF3, AF4, FPZ, FP1, and FP2. As shown in Figure 6, most of the selected channels came from the anterior hemisphere.

**Figure 7:**
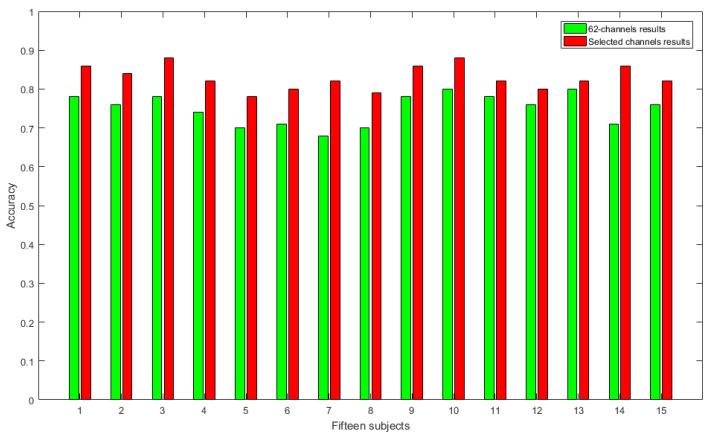
Classification results of positive and negative emotions.

**Figure 8:**
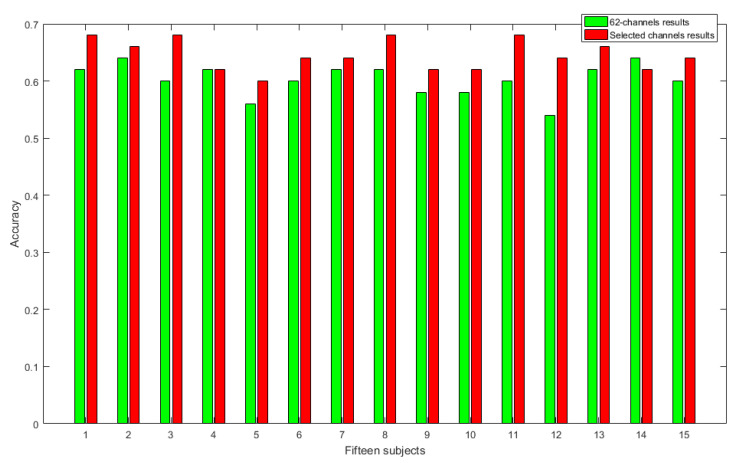
Classification results of positive, neutral, and negative emotions.

In order to evaluate the effectiveness of our proposed emotion recognition method, we compared the performances of the 62-channel and newly created 18-channel SEED datasets in classifying positive and negative emotions using CCE and NDE as a feature vector. Figure 7 shows the classification results for positive and negative emotions, while Figure 8 shows the classification results for positive, neutral, and negative emotions. The horizontal axis represents the 15 subjects, while the vertical axis represents the classification results. As shown in Figure 7, the 18 selected channels performed better in classifying positive and negative emotions, with a significant improvement in accuracy (*p* < 0.005) compared to the 62-channel dataset. For each subject in the dataset, the accuracy increases were 0.08, 0.09, 0.10, 0.08, 0.08, 0.09, 0.14, 0.08, 0.08, 0.09, 0.04, 0.06, 0.03, 0.14, and 0.07 in classifying positive and negative emotions (*p* < 0.005).

## 4. Discussion

This study proposed a method for fusing network entropy measures, used to achieve effective emotion recognition results based on EEG signals across subjects. The main innovations of the fused network entropy measures method are as follows: (1) mapping the time series of EEG signals to complex networks using the visibility graph method; (2) exploiting the CCE and NDE features from the complex network, describing the spatial properties of EEG signals in the form of local and global information, respectively; (3) using the cross-subject emotion training method based on the SEED dataset to overcome individual differences, thus improving the universality and generalizability of emotion recognition.

To illustrate the excellent performance of our method for emotion recognition, we compared our results with those of other studies based on same dataset, as well as a different dataset. We compared our work with the studies of Li et al. [7], Yucel et al. [29], Hao et al. [30], and Lu et al. [31], which also used the SEED dataset. In Li’s study, the positive and negative emotions in the EEG data collected from the SEED dataset were categorized. Eighteen linear and nonlinear EEG features (singular entropy, spectral entropy, permutation entropy, etc.) were extracted from the EEG signals. Then, these features were combined with the SVM classifier to categorize positive and negative emotions. The average value of each feature was used as the input of the support vector machine. In Li’s research, the framework of pattern learning used for emotion recognition was based on average entropy. In our work, the EEG dataset, classifier, and validation strategy were the same as in Li’s study, thus ensuring the fairness of performance comparison. In Yucel’s study, the convolutional neural network (CNN) architecture was exploited, whereby raw EEG data were used after applying windowing, preadjustments, and normalization. In Hao’s study, the raw feature vector sequence was extracted from multichannel EEG signals using a sliding window. The K-nearest neighbor algorithm was employed to estimate the emotion state. Lu et al. proposed dynamic entropy-based pattern learning with SVM to identify emotions from EEG signals, and then the positive and negative emotions in EEG data collected from the SEED dataset were categorized. Table 1 shows the accuracy comparison of the abovementioned methods from this study, as well as from the studies of Li et al., Yucel et al., Hao et al., and Lu et al., in the identification of negative and positive emotions among individuals. As shown in Table 1, the average accuracy of emotion recognition was 83.33%, 86.56%, 83.46%, and 85.11% using the methods from Li et al., Yucel et al., Hao et al., and Lu et al., respectively. However, in our work, we achieved an average accuracy of 87.26% based on complex network entropy measures. The experimental results show that our method performs well for emotion classification. The accuracy of cross-individual emotion classification was improved by 2.28% compared with other existing methods.

Furthermore, we compared our work with the studies of Yin et al. [32], Liu et al. [33], Asghar et al. [34], and Cheng et al. [35], who used different datasets. Yin et al. used a graph convolutional neural network and a long short-term memory neural network as the fusion model, named ERDL, to classify emotions. However, this method had the disadvantages of a long training time and tedious amounts of computation. Liu et al. exploited features from the time series and fused them to a vector for subject emotion recognition. However, this method only took into account the temporal information and left out the spatial information. Asghar et al. used gated recurrent units (GRUs) and recurrent neural networks (RNNs) to extract features from the SLGU-ENet model. Cheng et al. inputted features extracted from EEG signals into a deep forest classification model. Although their mean accuracy of emotion recognition was better than that in this study, the memory consumption was excessive and the model was bloated. As shown in Table 2, the average accuracy of emotion recognition was 84.81%, 84.30%, 82.60%, and 89.30% when using the methods from Yin et al., Liu et al., Asghar et al., and Cheng et al., respectively.

Differential entropy combined with SVM was used by Zheng et al. to achieve three categories (negative, positive, and neutral) of emotion recognition across subjects using the SEED dataset [6]. An average accuracy of 60.93% was obtained in Zheng’s study. An average accuracy of 64.15% was achieved in Lu’s research [31], which used a dynamic entropy-based pattern learning model to identify emotions from EEG signals. However, we achieved a higher average accuracy of 68.44% using network entropy measures of complex networks.

At present, using entropy measures as the input feature is one of the most effective methods for emotion recognition based on EEG signals. The human brain is a nonlinear dynamic system, and EEG signals have nonlinear and complex characteristics. Entropy measures are widely used to quantify the complexity of dynamic systems. A large amount of evidence has shown that clinically meaningful information can be effectively extracted from EEG signals using entropy measures. Using entropy measures and a machine learning classifier to recognize emotions based on EEG signals is an excellent method, as reported in various application cases. However, the entropy methods used in the current studies on emotion recognition of EEG signals lack spatial characteristics. By mapping EEG signals into complex networks and extracting the network structural entropy, we could not only effectively express the complexity of EEG signals, but also retain their spatial properties. The EEG signals are described in the two dimensions of time and space in order to obtain more features. Network structural entropy was used to create probability measures based on network parameters such as nodes and links. CCE and NDE represent the information of a complex network from local and global perspectives, respectively.

**Figure 9:**
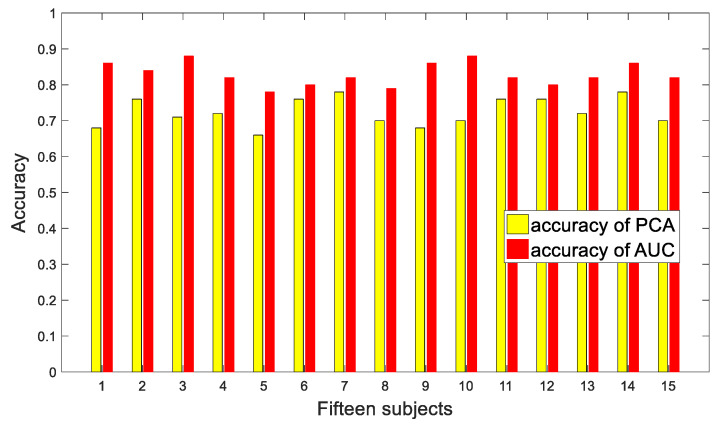
Classification results of PCA and AUC.

In order to determine the influences of different dimension reduction methods on our proposed emotion recognition method based on network entropy measures, we compared PCA, one of the currently popular methods, with AUC, which was adopted in our work. For the PCA method, the original data matrix was constructed from the data of 62 channels from the SEED dataset. Firstly, covariances among the data were calculated and sorted according to the eigenvalue of their covariance, from the largest to the smallest. The data with an eigenvalue of 0 were excluded, and the data with the lowest 50% eigenvalue were removed. The dimension reduction was completed by projecting the original data matrix onto the eigenmatrix corresponding to the selected eigenvalue. The classification results of the comparison are shown in Figure 9.

In our study, we proposed an effective emotion recognition method based on EEG signals, which achieved effective results. By measuring the entropy characteristics of the complex network structure, the spatial characteristics of EEG signals were increased. The EEG emotional patterns based on entropy measurements of complex networks were compared in detail with other existing studies, where it was shown that our method can effectively and accurately identify emotions.

Although excellent emotion classification accuracy was obtained in our work, there remains room for improvement. First, we will determine the interpretability aspects of complex networks associated with brain networks. Moreover, we will explore other advanced machine learning methods, such as deep neural networks and deep learning, to investigate emotion recognition across individuals.

## 5. Conclusions

In our work, we exploited network entropy measures with the SVM classifier to implement an excellent model for emotion recognition based on EEG signals. We mapped the EEG signals to complex networks and extracted the network entropy measures to represent the temporal and spatial characteristics of EEG information. We conducted a classification study on three emotions: positive, neutral, and negative. In order to improve the universality of our approach, we used the cross-subject emotion training method based on complex networks to overcome individual differences and build a more robust emotion recognition method. After reducing the dimensionality of features using the AUC method, effective features were fused to a feature vector input into a support vector machine to recognize emotions. In addition, we carried out a detailed comparison between our work and existing studies. The results fully prove that emotion recognition utilizing network entropy measures of complex networks can achieve better results. Owing to its excellent generalizability, our proposed emotion recognition method has great potential applicability in brain–computer interfaces.

## Figures and Tables

**Table 1:** Accuracy comparison of methods from this study, Yucel et al., Hao et al., Lu et al., and Li et al. in the identification of positive and negative emotions across individuals from the SEED dataset.

	Dataset	Year	Methodology	Cross-Subject	VS	Classifier	Mean Accuracy	StdACC
Zhang’s study [36]	SEED	2021	DE	Yes	LOSO	ResNets	86.43%	—
Yucel’s study [29]	SEED	2020	IR-V2	Yes	LOSO	CNN	86.56%	6.94%
Hao’s study [30]	SEED	2020	DBN-CRF	Yes	LOSO	KNN	83.46%	—
Lu’s study [31]	SEED	2019	DySampEns	Yes	LOSO	SVM	85.11%	11.54%
Li’s study [7]	SEED	2018	ApEn, ShEn, etc.	Yes	LOSO	SVM	83.33%	10.16%
Our work	SEED	2021	NEM	Yes	LOSO	SVM	87.26%	6.06%

SVM: support vector machine; CNN: convolutional neural network; KNN: K-nearest neighbor; NEM: network entropy measures; VS: validation strategy; LOSO: leave-one-subject-out; StdACC: standard deviation of accuracy; CN: complex networks; DBN-CRF: deep belief network with conditional random field; IR-V2: InceptionResnetV2; StdACC: standard deviation of accuracy; DySampEns: dynamic sample entropies; ApEn: approximate entropy; ShEn: Shannon entropy, DE: differential entropy.

**Table 2:** Accuracy comparison of methods from this study, Yin et al., Liu et al., Asghar et al., and Cheng et al. in the identification of positive and negative emotions using various datasets.

	Dataset	Year	Methodology	Cross-Subject	Classifier	Mean Accuracy	StdACC
Yin’s study [32]	DEAP	2021	DEAP	YES	GCN, LSTM	84.81%	—
Liu’s study [33]	DEAP	2021	2021	No	SVM	84.30%	—
Asghar’s study [34]	DEAP	2021	No	No	SVM	82.60%	6.54%
Cheng’s study [35]	DREAMER	2021	SVM	No	DF	89.03%	5.56%
Our work	SEED	2021	84.30%	Yes	SVM	87.26%	6.06%

DE: differential entropy; SVN: support vector network; DF: deep forest; GCN: convolutional neural network; LSTM: long short-term memory neural network; SVM: support vector machine.

## Data Availability

Publicly available datasets were analyzed in this study. This data can be found here: [https://bcmi.sjtu.edu.cn/~seed/seed.html].
